# miR-22 alleviates sepsis-induced acute kidney injury via targeting the HMGB1/TLR4/NF-κB signaling pathway

**DOI:** 10.1007/s11255-022-03321-2

**Published:** 2022-08-12

**Authors:** Jie Zhang, Qi Chen, Zhuquan Dai, Huibin Pan

**Affiliations:** 1 Emergency Intensive Care Unit, The First People’s Hospital of Huzhou, 158 Guangchanghou Road, Huzhou, 313000 Zhejiang China; 2 Department of Nephrology, The First People’s Hospital of Huzhou, Huzhou, 313000 Zhejiang China

**Keywords:** microRNA, Sepsis, Acute kidney injury, HMGB1, Signaling pathway

## Abstract

**Background:**

Acute kidney injury (AKI) is a severe complication of sepsis, and is strongly correlated with MicroRNAs (miRNAs). However, the mechanism of miR-22 on sepsis-induced AKI is not clearly understood. The study aimed to explore the role and mechanism of miR-22 on AKI.

**Methods:**

The AKI models were established by cecal ligation and puncture (CLP) surgery in SD rats and lipopolysaccharide (LPS) induction in HBZY-1 cells. In AKI rats, the content of serum creatinine (SCr) and blood urea nitrogen (BUN) were detected. Kidney tissues were pathologically examined by H&E and PAS staining. The LPS-induced HBZY-1 cells were transfected with mimics miR-22, si-HMGB1, or oe-HMGB1. miR-22 and HMGB1 expression was detected in vivo and in vitro. In transfected cells, HMGB1/TLR4/NF-κB pathway-related protein expressions were measured by Western blot. The relationship between miR-22 and HMGB1 was assessed by a dual-luciferase gene report. Inflammatory cytokine levels in serum and cells were assessed by ELISA.

**Results:**

In AKI rats, kidney injury was observed, accompanied by the down-regulated miR-122 expression and up-regulated HMBG1 expression. The dual-luciferase report found miR-22-3p could targetly regulate HMBG1. Furthermore, both in vitro and in vivo experiments revealed that the releases of inflammatory cytokine were increased after AKI modeling, but the situation was reversed by mimics miR-22 or si-HMGB1 in vitro. In HBZY-1 cells, mimics miR-22 could suppress LPS-induced overexpression of HMGB1/TLR4/NF-κB signaling pathway-related proteins. However, the oe-HMGB1 addition reversed the effect of mimics miR-22.

**Conclusion:**

miR-22 can inhibit the inflammatory response, target the HMGB1, and inhibit the HMGB1/TLR4/NF-kB pathway, to attenuate the sepsis-induced AKI, which indicates that miR-22 may serve as a potential treatment target in sepsis-induced AKI.

## Introduction

Sepsis is a kind of heterogeneous syndrome that can lead to various organ disorders, such as kidney [1]. Acute kidney injury (AKI) is a common disease mainly manifested by a sharp decline in renal function [2]. These days, although the treatment of AKI has improved, the morbidity and mortality of AKI are still high [3]. Sepsis is not only a major complication of AKI, but also a common cause of AKI [4]. The pathological mechanism of sepsis-induced AKI is complex, involving numerous factors, such as altered kidney hemodynamics, endothelial cell disorder, as well as excessive inflammation [5, 6]. Among these factors, the early inflammatory response of the kidney is often identified as the main AKI mechanism [7]. Published reports have found that during the occurrence of sepsis, pathogens can stimulate the body to secrete massive inflammatory factors to kidneys, and the inflammatory factors are recognized by the Toll-like receptors (TLRs) of renal tubular epithelial cells [8, 9].

High mobility group box1 (HMGB1), a widely available protein, is often combined with DNA as a structural protein of chromatin [10]. HMGB1 mainly exists in the nucleus and participates in the maintenance of nucleosome morphology and the replication of DNA [11]. HMGB1 can activate the pro-inflammatory signal pathway by binding with receptors like TLR2 and TLR4 [12]. As a potential inflammatory cytokine, HMGB1 is closely associated with many kidney diseases. Accumulating evidence has revealed that the expression of HMGB1 in the blood, urine, kidney tissue, cytoplasm, and extracellular matrix is increased in patients with kidney disease [13, 14].

MicroRNA (miRNA), a non-coding RNA only containing 18–25 nucleotides, is an endogenous regulator of the target gene that plays a crucial role in anti-inflammatory and repairing damaged cells [15, 16]. Functionally, miRNAs participate in various biological processes [17]. Some studies have reported that miR-22 is strongly correlated with sepsis-induced diseases and AKI [18]. For example, miR-22 can alleviate sepsis-induced cardiomyopathy by regulating Sirt1 [19]. Furthermore, down-regulated miR-22 expression in AKI patients can be used as a biomarker to predict AKI occurrence [20]. Besides, it has also been found that miR-22 can targetly regulate HMGB1. For instance, miRNA-22 can inhibit retinoblastoma cell viability, migration and invasion by regulating HMGB1 [21]. miR-22 inhibits arterial smooth muscle cell proliferation and migration by targeting HMGB1 in arteriosclerosis obliterans [22]. Nevertheless, there remain rare relatively studies regarding the role of miR-22 and HMGB1 in sepsis-related AKI.

Hence, in this study, we induced an animal model of sepsis-related AKI by cecal ligation and puncture (CLP) method and used LPS to construct the AKI cell model to evaluate the role of miR22 and HMGB1 in sepsis-related AKI, providing a scientific basis for further development of miR-22 as a novel therapeutic target for treating AKI.

## Materials and methods

### Experimental animals

Sprague Dawley rats (6 weeks old, weighing 200–230 g) were provided by the SLAC Laboratory Animal Co., Ltd (Animal License No: SCXK Hu 2017–0005). The rats were raised in 12/12 h light/dark conditions with 60 ± 10% humidity and 20 ± 2 ℃, and acclimatized for 7 days before experiments. During this period, all rats were free to eat food and drink water. Our research was ratified by the approval of the Animal Experimentation Ethics Committee of Zhejiang Eyong Pharmaceutical Research and Development Center (Certificate No. SYXK (Zhe) 2021–0033), and the experimental operation was based on the guidelines for the use of animal care.

### CLP sepsis model

Twelve Sprague Dawley rats were randomly classified into the sham group and sepsis group. Sepsis modeling was conducted by a CLP method as previously described [23]. Briefly, rats in the sepsis group were anesthetized with isoflurane, then a 20 mm incision was cut from the middle of the abdominal wall, and the cecum was pulled out gently, avoiding damage to mesenteric vessels. Then, the midpoint of the cecum was connected to the ileocecal valve with a silk thread for ligation, and the cecum was punctured twice at the ligation site with a 21 G sterile needle. Next, the cecum was gently compressed to extrude small quantity of fecal contents through the puncture site. Upon the extruding fecal contents were wiped, the cecum was pushed back to the abdominal cavity and the incision was sewed. In the sham group, there was no cecal ligation or puncture, and the other operations were the same as those in the sepsis group. After surgery, rats received 5 mL/100 g of 37 ℃ saline for saline and 0.05 mg/kg of buprenorphine for pain relief. The body characteristics of the rats were observed every hour to ensure the success of modeling. After 24 h of the surgery, the rats were anesthetized with 3% pentobarbital podium and euthanized. The kidney tissues and blood samples were taken out, then, the blood was centrifuged at 10,000 rpm for 10 min at 4 ℃, and the kidney tissues and blood supernatant were stored in the refrigerator at − 80 ℃ for standby.

### Biochemical assay

The serum creatinine (SCr) and blood urea nitrogen (BUN) contents were measured with a biochemical instrument based on the operation instructions.

### Histopathological analysis of kidney

To perform the histopathological analysis of kidney tissues in each group, H&E and PAS staining were used following general protocol. In brief, kidney tissues were removed and fixed in paraformaldehyde solution. Then the tissue samples were dewaxed in xylene, rehydrated with ethanol, and stained with H&E and PAS staining (Servicebio, G1003; Servicebio, G1008) respectively. The slices were observed and took photos with an optical microscope (× 100 and × 400 magnification; Nikon, E100).

### Double luciferase gene reporter analysis

Through double luciferase gene technology, we found there was a binding site between HMBG1 and miR-22. For luciferase reporter assay, the sequences covering the miR-22 target site in the wild-type (WT) and the mutant (MUT) 3’ UTR of HMBG1 were cloned into the pGL3 promoter vector. HBZY-1 cells were co-transfected with WT-HMBG1 (or MUT-HMBG1) and miR-22 (or miR-NC) with Lip 3000 (Thermo Fisher Scientific). After culturing for 48 h, the relative activity of firefly luciferase was analyzed by dual‐luciferase reporter gene assay kit (Solaibao, D0010-100 T).

### Cell culture and transfection

Rat glomerular mesangial cells (HBZY-1) were purchased from icell bioscience Inc. (iCell-r013) and cultured in deme high glucose medium containing 10% fetal bovine serum, 100 U/ml penicillin and 100 U/ml streptomycin in 5% CO_2_ incubator at room temperature. Then the cells were seeded onto 24-well plates (5 × 10^5^ cells/well) and divided into seven groups: (1) Control, (2) LPS, (3) LPS + mimics NC, (4) LPS + mimics miR-22, (5) LPS + si-NC, (6) LPS + si-HMGB1, (7) LPS + oe-HMGB1. After the concentration of the cells reached 80%, all the HBZY-1 cells were transfected with mimics miR-22, mimics NC, si-HMGB1, oe-HMGB1, si-NC (GenePharma) for 6 h according to the grouping. Next, except for the control group, all cells were exposed to LPS (10 μg/m) for 12 h to induce AKI. After the transfection, the cell suspension was centrifuged at 800 g/min for 10 min, collected the supernatant and then filtered it with 0.22 μM microporous membrane filtration, collected the filtration solution, and stored it at – 40 ℃ for the following experiments.

### Quantitative PCR (q-PCR)

The total RNA in the kidney tissue or cells was extracted by Trizol reagent (Sangon, B511311). cDNA was synthesized by RNA reverse-transcription kit (CWBIO, CW2569). Hereafter, the q-PCR was conducted with SYBR Premix Ex TaqII (Takara, RR820A). GAPDH was taken as a reference. 2^−ΔΔCt^ method was used to determine the expression of relative genes. The list of primers used in this study was shown in Table 1.

**Table 1:** qRT-PCR primer sequence

Gene	Forward Primer	Reverse Primer
Rat miR-22	TGAGCCGCAGTAGTTCTTCAG	GGCAACAGTTCTTCAACTGGC
Rat HMGB1	CCTGCATATTGTGGTAGGGGT	GGCAAAGGCATTACAGCCAG
Rat GAPDH	TGTGAACGGATTTGGCCGTA	GATGGTGATGGGTTTCCCGT

### Western blot analysis

The total protein of rat kidney tissue and HBZY-1 cells were lysed on ice with pre-cooled RIPA buffer (Biyuntian, P0013D), and assessed the concentration by the bicinchoninic acid (BCA) protein assay kit (Solarbio, pc0020). The same amount of protein was separated by 12% SDS-PAGE. Then, the protein was transferred to the PVDF membranes. After being blocked at 37 ℃ for 2 h with 5% skim milk, the membranes were incubated overnight at 4 ℃ with primary antibodies against HMGB1 (1:1000, AF7020), TLR4 (1:1000, AF7017), TLR2 (1:1000, DF7002), MyD88 (1:1000, AF5195), TM (1:1000, DF6291), NF-κB p65 (1:1000, AF5002) and p-NF-κB p65 (1:1000, AF2006). An antibody against GAPDH (1:5000, AF7021) was used as the loading control. The membranes were then washed and incubated with horseradish peroxidase-conjugated secondary antibodies at 37 ℃ for 1 h. Finally, the membranes were washed and developed by ECL. The densitometry analysis of the immunoreactive bands was performed by the Fuji ultrasonic-Doppler velocity profile system and Image J program. All the primary antibodies were purchased from Affinity.

### Enzyme-linked immunosorbent assay (ELISA)

Took the frozen rat serum samples and HBZY-1 cell filtered solution, and then ELISA Kits were applied to measure the content of inflammatory cytokines, including TNF-α (Meimian, MM-0132M1), IL-1β (Meimian, MM-0040M1), IL-6 (Meimian, MM-0163M1) and TM (Meimian, ml-091721). Calculating the inflammatory factors’ expression levels according to the standard sample.

### Statistical analysis

The data of the study were presented as mean ± SD, and analyzed by SPSS 20.0. One-way ANOVA and SNK tests were applied for multi-group comparison. Kruskal–Wallis *H* test was applied if variances were not equal. *p* < 0.05 was considered a statistically significant difference.

## Results

### Sepsis significantly induced AKI in vivo

**Fig. 1:**
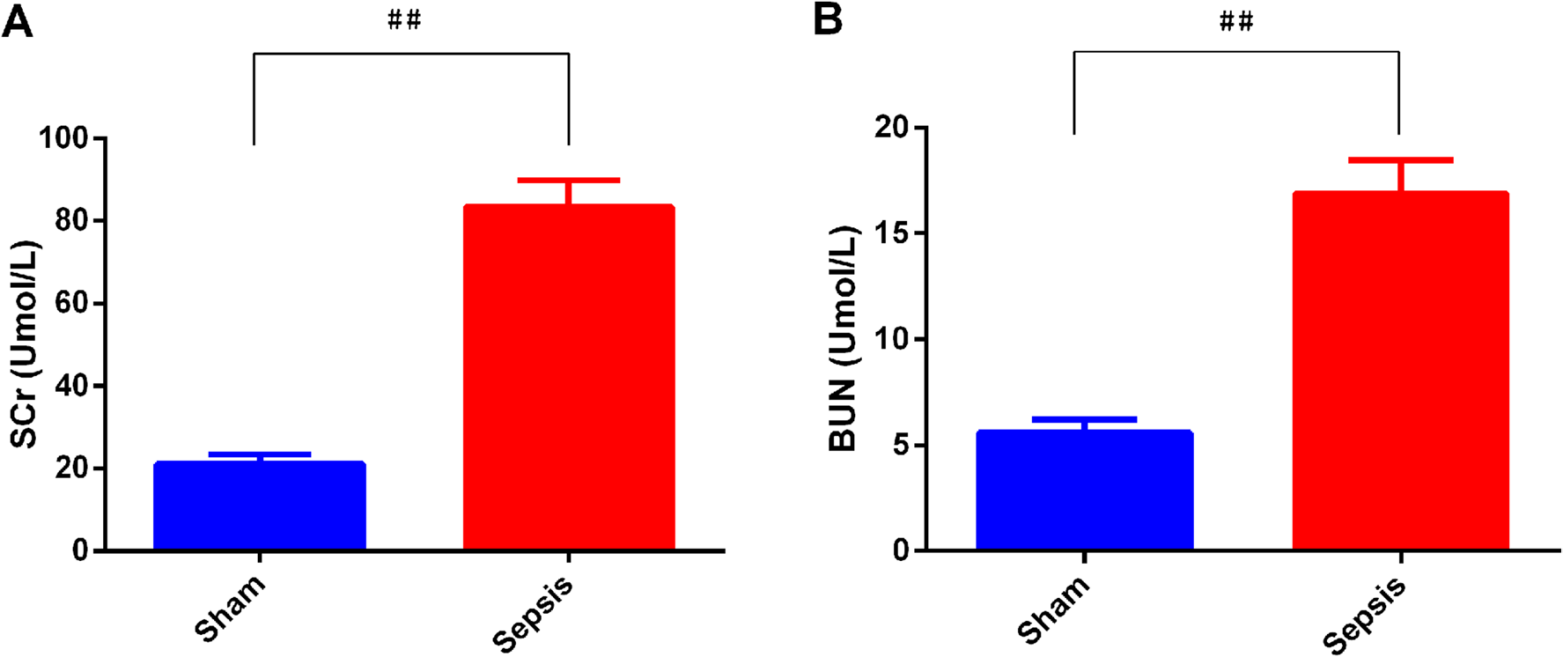
The contents of serum creatinine (SCr) and blood urea nitrogen (BUN) were detected in cecal ligation and puncture (CLP)-induced acute kidney injury (AKI) rats. The contents of SCr (**A**) and BUN (**B**) were compared between the sham group and the sepsis group. ^#^*p* < *0.05*, ^##^*p* < *0.01* vs. Sham. *n* = 6

As presented in Fig. 1, after 24 h of the surgery, the contents of SCr and BUN increased significantly in the sepsis group compared to those in the sham group (*p* < 0.01).

**Fig. 2:**
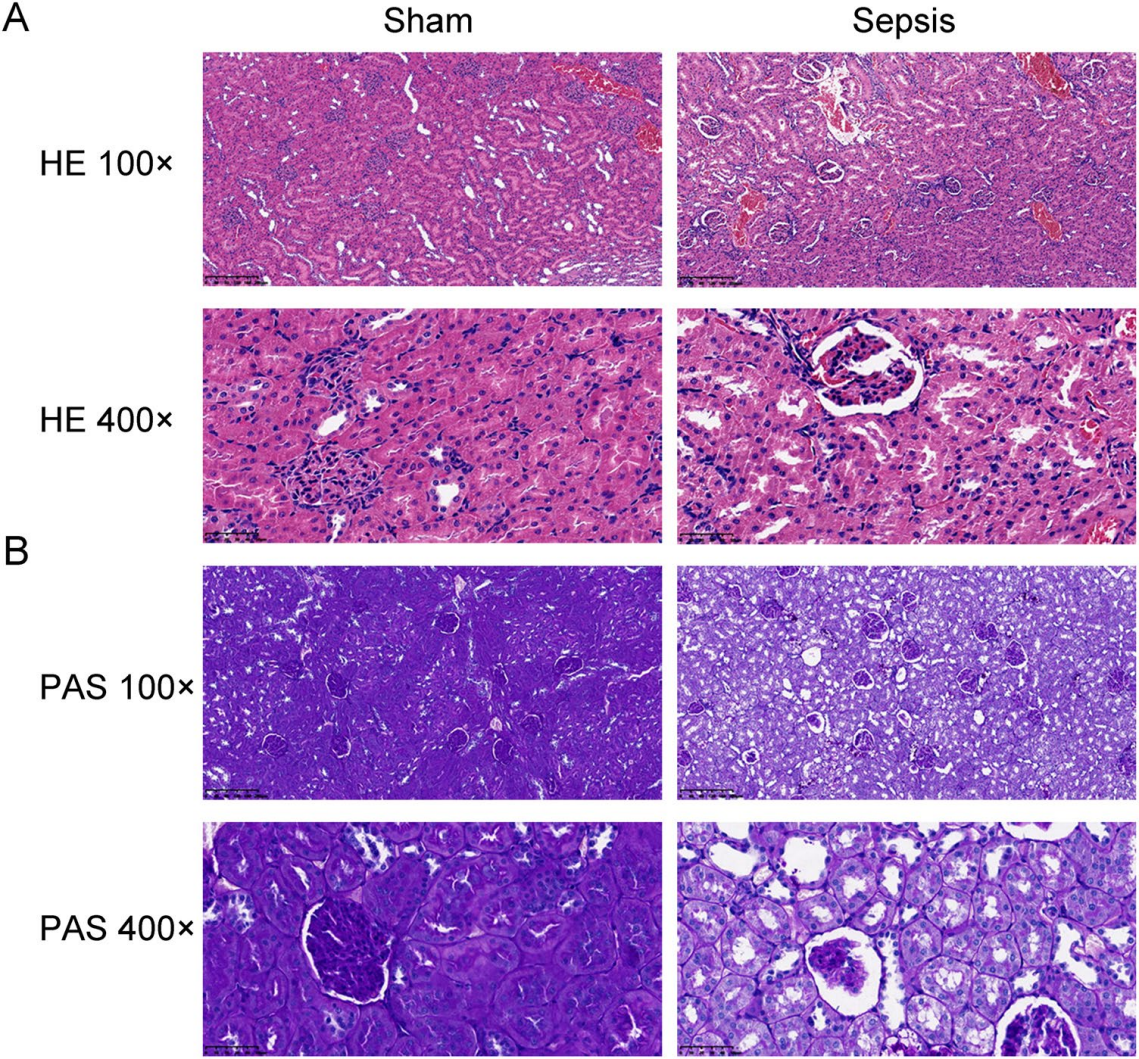
The kidney histopathological structure was damaged in CLP-induced AKI rats. Hematoxylin and eosin (H&E, A) and periodic acid-schiff (PAS, B) were used to perform histopathological analysis of kidneys in sham and sepsis groups. Original magnification 100 × or 400 ×

As exhibited in Fig. 2, HE and PAS staining of the kidney tissues showed that the glomerulus, extracellular matrix and tubules were all normal and clear in the sham group. However, mass abnormalities were observed in the sepsis group, sepsis rats developed serious histological kidney injuries including glomerular basement membrane thickened, inflammatory cell infiltration, as well as glomerular ruptured.

### miR-122 was down-regulated while HMBG1 was up-regulated in AKI model rats

**Fig. 3:**
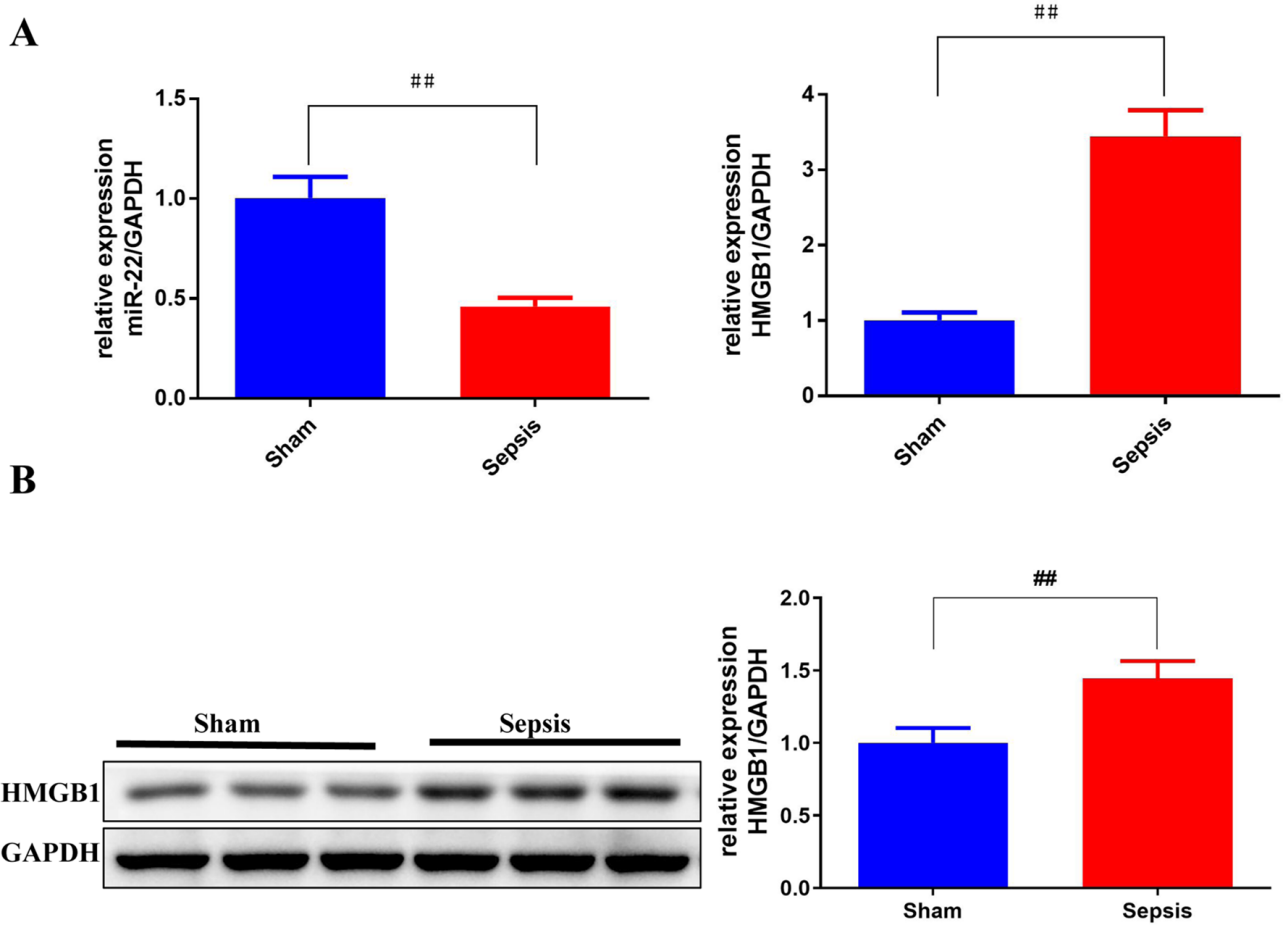
The expression of miR-22 was down-regulated and the expression of HMGB1 was up-regulated in CLP-induced rats. Q-PCR (quantitative PCR) was conducted to detect the expression of miR-22 and HMGB1 mRNA in rats (**A**) and western blot was performed to detect the expression of HMGB1 protein in rats (**B**). ^#^*p* < *0.05*, ^##^*p* < *0.01* vs. Sham. *n* = 6

As shown in Fig. 3, relative to the sham group, the miR-22 mRNA expression was notably down-regulated (*p* < 0.01), while the HMGB1 mRNA and protein expression were obviously up-regulated in the sepsis group (*p* < 0.01).

### miR-22-3p could targetly regulate HMBG1

**Fig. 4:**
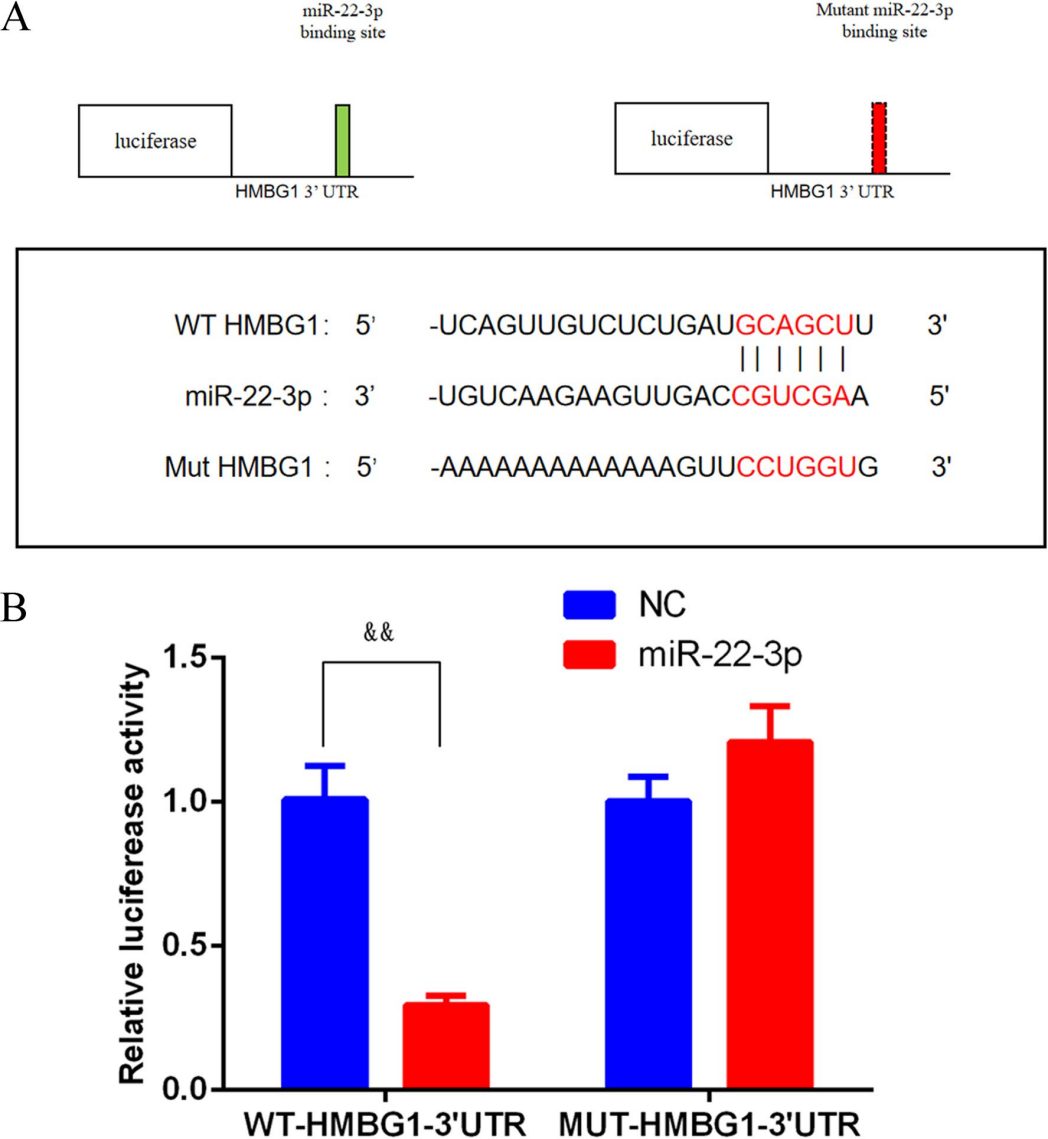
HMGB1 was a direct target of miR-22. Double luciferase gene reporter analysis showed that there was a binding site between miR-22 and the 3’-UTR of HMBG1 mRNA (**A**). The luciferase activity was assessed to approve the relationship between HMGB1 and miR-22 (**B**). ^*&&*^*p* < *0.01 *vs. NC

The dual-luciferase report revealed that the miR-22-3p target sequence was located at 1162-1169 bp of WT-3’-UTR of the HMBG1 gene, and MUT-HMBG1-3’-UTR was not the target site of miR-22-3p (Fig. 4A). Subsequently, the result of the dual-luciferase report demonstrated that overexpression of miR-22-3p notably decreased the luciferase activity of WT-HMBG1 (*p* < 0.01), but did not influence the luciferase activity of MUT-HMBG1 (*p* > 0.05, Fig. 4B). All of these indicated that miR-22-3p could target HMBG1 directly.

### miR-22 suppressed the content of inflammatory cytokines in vivo and in vitro

**Fig. 5:**
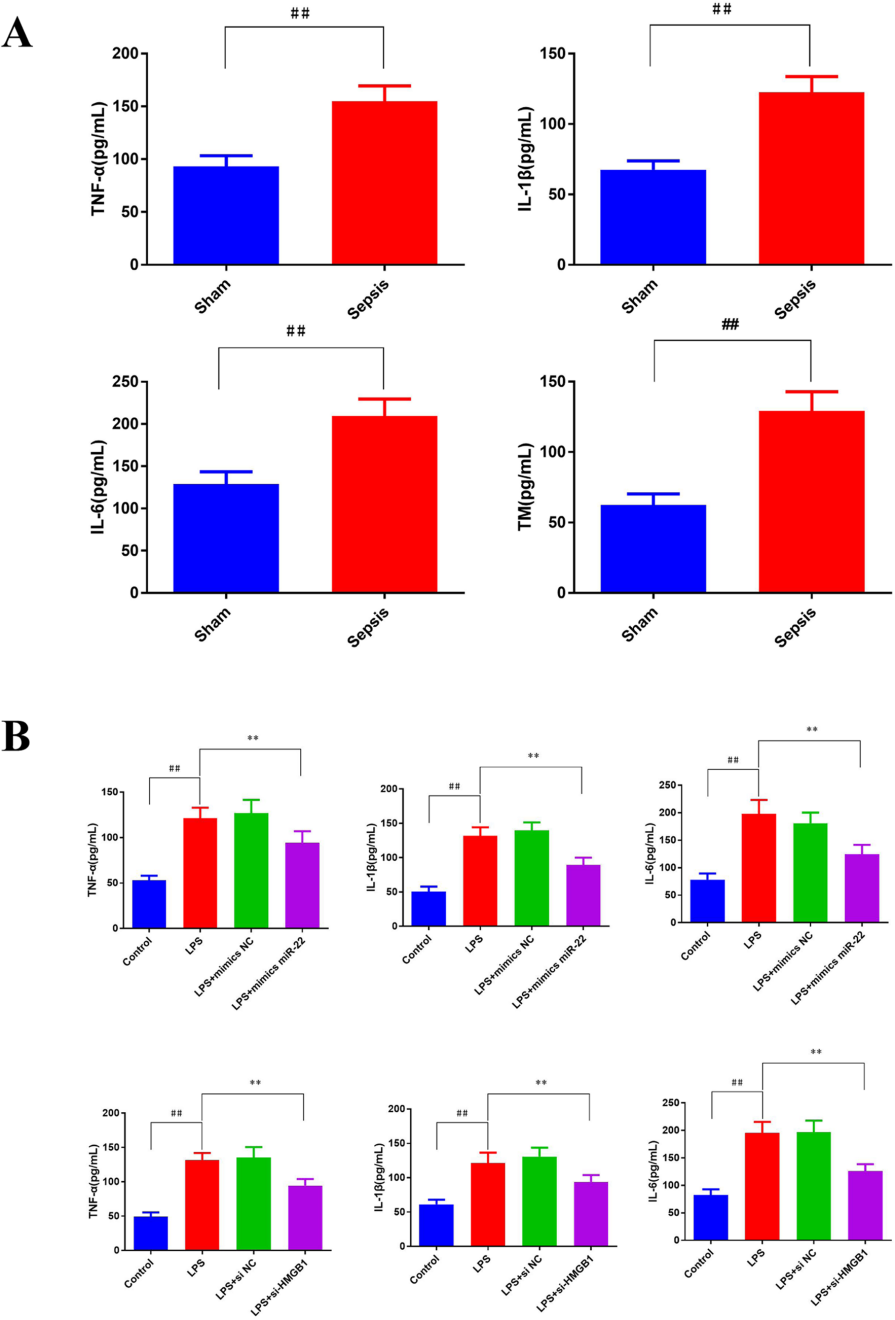
miR-22 attenuated LPS-induced inflammatory response in the AKI model. ELISA was applied to detected the content of inflammatory factors in model rats (**A**) and HBZY-1 cells (**B**), ^#^*p* < *0.05*, ^##^*p* < *0.01 vs.* Sham or control; *p < *0.05*, **p < *0.01 vs.* lipopolysaccharide (LPS)

To investigate the relationship among miR-22, HMGB1 and the inflammatory cytokines in the models of AKI in vivo and in vitro, ELISA was conducted. As showcased in Fig. 5A, in vivo experiment, the content of TNF-α, IL-1β, IL-6 and TM in the sepsis group was significantly higher than those of the sham group (*p* < 0.01).

The changes of inflammatory cytokine contents in vitro were similar to those observed in vivo. Compared with the control group, the content of TNF-α, IL-1β, and IL-6 was increased remarkably in the LPS group (*p* < 0.01). Nevertheless, transfection with si-HMGB1 or mimics miR-22 significantly reduced LPS-induced inflammatory cytokine content (Fig. 5B).

### miR-22 suppressed the protein expression of HMGB1 in AKI vitro model

**Fig. 6:**
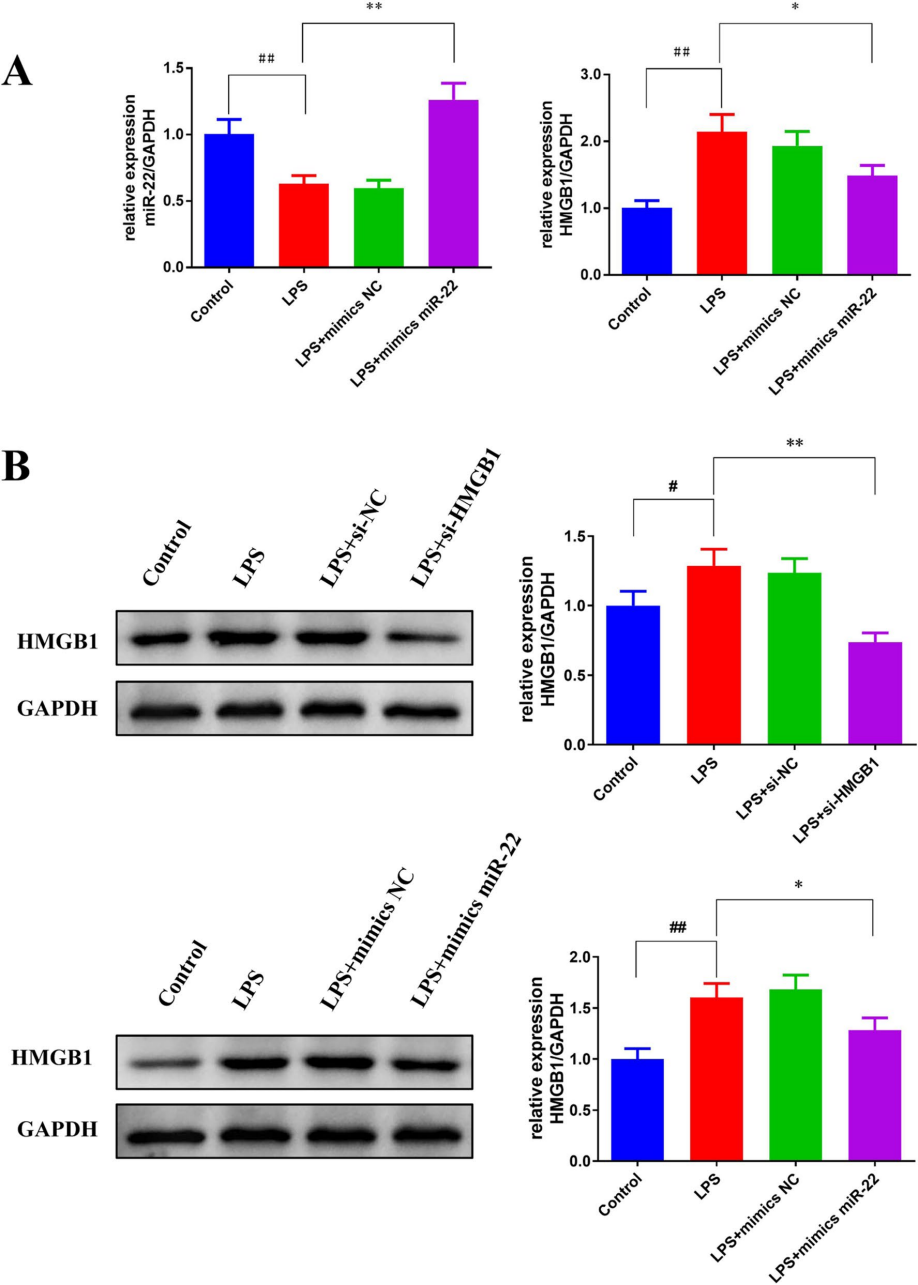
miR-22 inhibited HMGB1 protein expression in AKI vitro model. Q-PCR was applied to measure the expression of miR-22 and HMGB1 mRNA in HBZY-1 cells (**A**) and western blot was used to detect the expression of HMGB1 protein in HBZY-1 cells (**B**), ^#^*p* < *0.05*, ^##^*p* < *0.01 *vs. Control; *p < *0.05*, **p < *0.01 *vs. LPS

As illustrated in Fig. 6A, LPS stimulation led to a significant down-regulation of miR-22 mRNA expression and a significant up-regulation of HMGB1 mRNA expression in HBZY-1 cells (*p* < 0.05). However, the expression of miR-22 and HMGB1 mRNA were reversed by the transfection of mimics miR-22 in LPS-induced HBZY-1 cells.

To further determine the relationship between miR-22 and HMGB1, western blot was applied to analyze the expression of HMGB1 protein. Compared with the control group, the expression of HMGB1 protein was significantly increased in the LPS group (*p* < 0.05), whereas transfection with si-HMGB1 or mimics miR-22 significantly reduced HMGB1 protein expression, and the effect of miR-22 on HMGB protein expression was similar to HMGB inhibitor (Fig. 6B).

### miR-22 regulated the expression of HMGB1/TLR4/NF-κB signaling pathway-related proteins

**Fig. 7:**
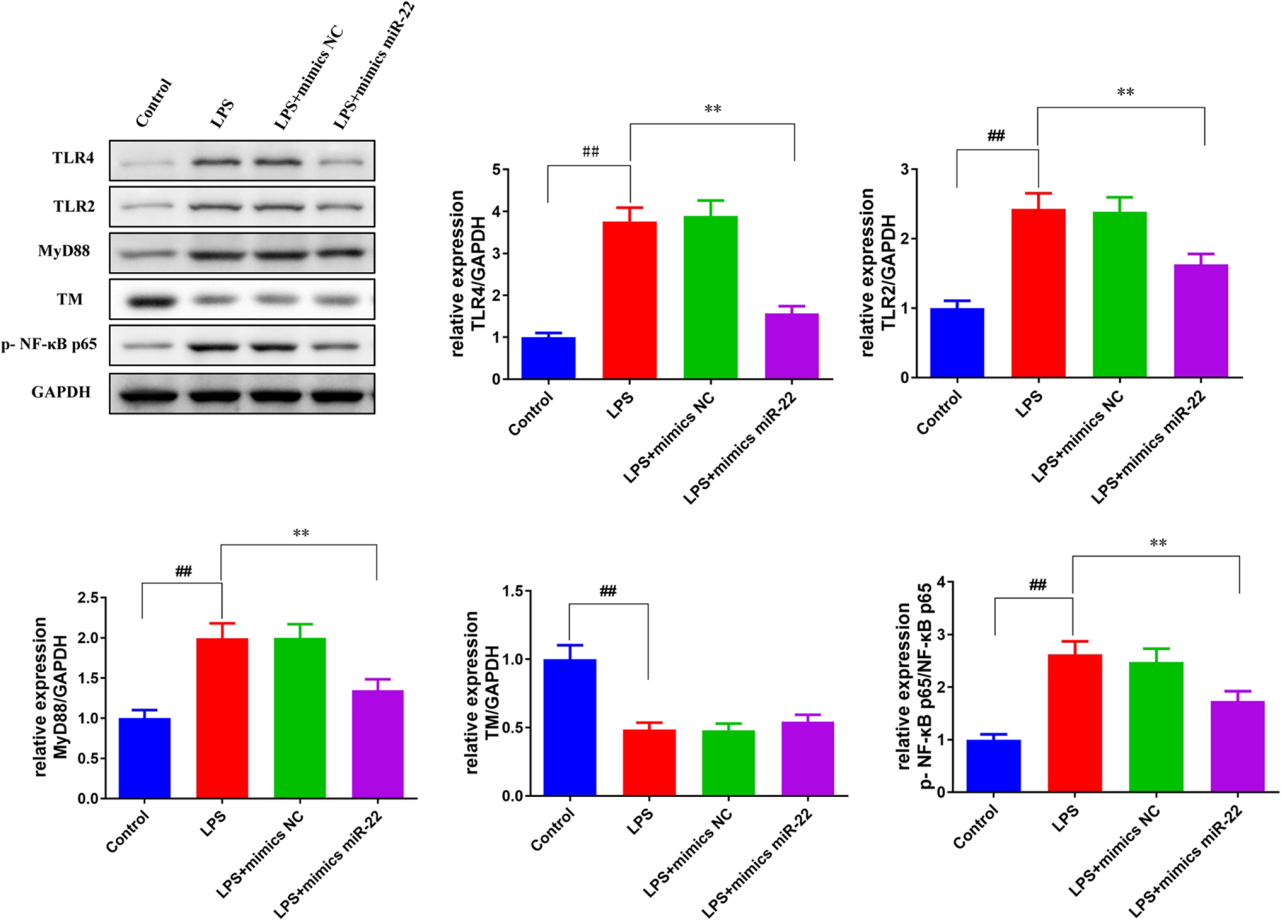
miR-22 decreased the expression of TLR4, TLR2, MyD88, and p-NF-κB p65 protein in the AKI vitro model. Western blot was conducted to measure the protein expression of TLR4, TLR2, MyD88, TM and p-NF-κB p65 in HBZY-1 cells. ^#^*p* < *0.05*, ^##^*p* < *0.01 vs.* Control. *p < *0.05*, **p < *0.01 vs.* LPS

**Fig. 8:**
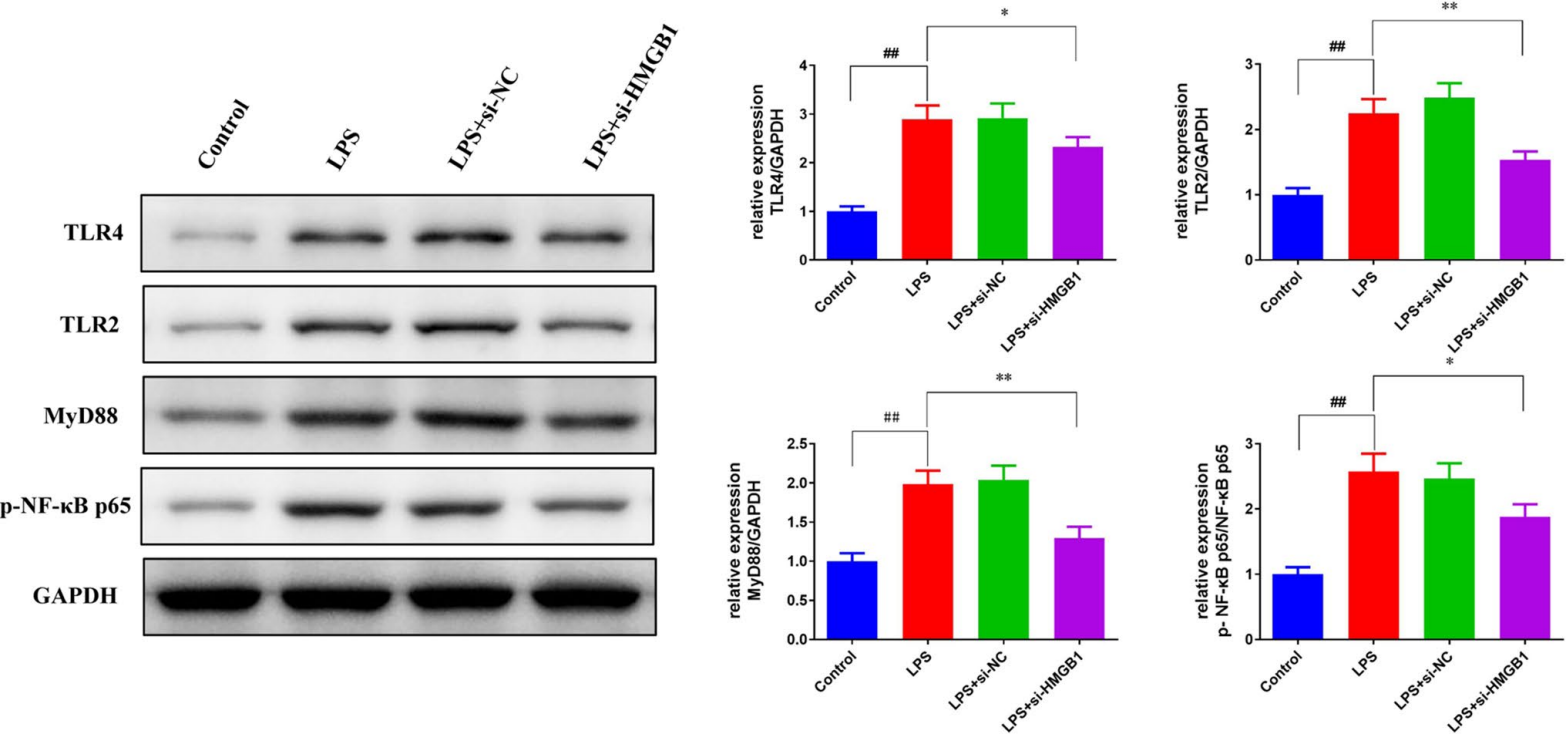
si-HMGB1 decreased the expression of TLR4, TLR2, MyD88, and p-NF-κB p65 protein in the AKI vitro model. Western blot was used to measure the protein expression of TLR4, TLR2, MyD88, and p-NF-κB p65 in HBZY-1 cells. ^#^*p* < *0.05*, ^##^*p* < *0.01 vs.* Control. *p < *0.05*, **p < *0.01 vs.* LPS

**Fig. 9:**
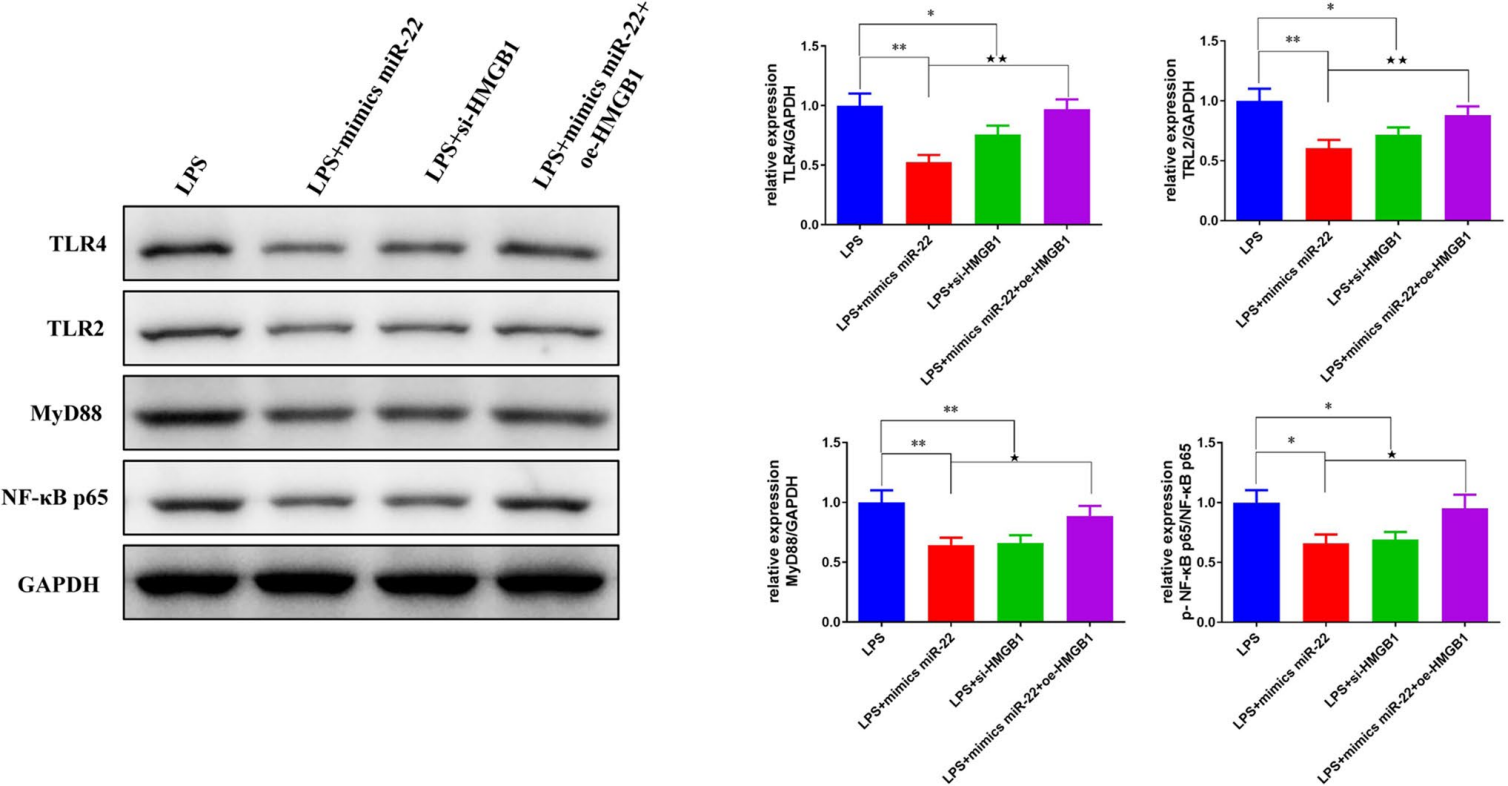
miR-22 alleviated AKI by targeting the HMGB1/TLR4/NF-κB signaling pathway. Western blot was carried out to measure the protein expression of TLR4, TLR2, MyD88, and p-NF-κB p65 in HBZY-1 cells. *p < *0.05*, **p < *0.01 vs.* LPS, ^*★*^*p* < *0.05*, ^*★★*^*p* < *0.01 vs.* LPS + mimics miR-22

To reveal the underlying mechanism of miR-22 in LPS-induced AKI, we conducted western blot to measure TLR4, TLR2, MyD88, and p-NF-κB p65 protein expression. Compared with the control group, the TLR4, TLR2, MyD88, p-NF-κB p65 protein expression in the LPS group were significantly up-regulated (*p* < 0.01*,* Figs. 7 and 8), while the TM protein expression was significantly down-regulated (*p* < 0.01). Besides, the increased or decreased expression of these proteins induced by LPS was partly counteracted by mimics miR-22 or si-HMGB1 transfection (Figs. 7 and 8). However, TLR4, TLR2, MyD88 and p-NF-κB p65 protein expression inhibited by miR-22 in LPS + mimics miR-22 group were notably up-regulated through the addition of oe-HMGB1 in the LPS + mimics miR-22 + oe-HMGB1 group (Fig. 9).

## Discussion

In the present study, the CLP method was performed to induce AKI in rats, which was characterized by the notable up-regulation of SCR, BUN contents, and serum inflammatory cytokine release, as well as the obvious damage in the kidney histopathological structure. In addition, we confirmed that miR-22 was remarkably low-expressed while HMGB1 was obviously high-expressed in AKI models. The luciferase report found that miR-22 could targetly regulate HMGB1. Furthermore, the elevated contents of inflammatory cytokines induced by LSP were decreased by mimics miR-22 or si-HMGB in vitro. The results of q-PCR and Western blot demonstrated that miR-22 could target HMGB1 and affect the expression of TLR4, TLR2, MyD88, p-NF-κB p65 protein. All of those indicated that miR-22 could alleviate sepsis-related AKI via targeting the HMGB1/TLR4/NF-κB signaling pathway.

MicroRNAs (miRNAs), short-chain non-coding RNAs, are important for gene expression, various miRNAs have been indicated involved in many human diseases [24]. For example, increasing the expression of miR-125b can improve sepsis-induced cardiac dysfunction and enhance the survival rate in mice [25]. Furthermore, Liu et al. conducted an in vivo experiment and found that miR-155 can decrease inflammation response in septic lung injury [26]. A similar study also has reported that miR-590-3p can alleviate AKI induced by LPS as well as inhibit podocyte apoptosis [27]. In addition, it is reported that miR-22 can suppress kidney fibrosis, which indicated that miR-22 might offer a pivotal approach to the treatment of renal injury [28]. Hence, we hypothesize that miR-22 is associated with sepsis-induced AKI. In this research, we found that transfection of mimics miR-22 in LPS-induced HBZY-1 cells could effectively decrease the expression of HMBG1, double luciferase gene reporter analysis also proved that miR-22 could targetly regulate HMBG1.

HMGB1 is not only an early inflammation mediator but also plays an important role as a late mediator of lethal sepsis [29]. Previous studies have demonstrated that HMGB1 is involved in the acute inflammatory response and contributes to the injuries [30, 31]. It has been reported by Entezari et al. that suppressing HMGB1 expression can attenuate the inflammatory response in acute lung injury induced by hypoxia [32]. Kim et al. also found that HMGB1 is massively released during the acute damaging period in the postischemic brain induced by NMDA [33]. Furthermore, increased HMGB1 was observed in kidney transplantation, kidney ischemia reperfusion injury and other kidney injuries [34]. Based on these theoretical foundations, we hypothesized that overexpressed HMGB1 was disadvantageous, for it could up-regulate the contents of inflammatory cytokines. In LPS-induced AKI, inflammation is one of the primary causes for acute kidney disorder [35]. In this study, the content of inflammatory factors and the expression of HMGB1 were decreased after LPS-induced cells transfected with mimics miR-22 or si-HMGB1, which indicated that decreased HMGB1 may be a promising approach for the attenuation of LPS-induced inflammation.

HMGB1/TLR4/NF-κB signaling pathway is critical in the occurrence and development of inflammatory responses [36]. TLR2 can recognize a wide variety of pathogens and is responsible for the inflammatory cascade in sepsis [37]. TLR4 can increase MyD88 expression, activate the NF-κB signaling pathway and induce the release of inflammatory factors [38]. HMGB1 is an endogenous cell secreted ligand for TLR2/4, binding of HMGB1 to TLR2 will promote a MyD88-dependent signaling cascade and activate NF-κB, and eventually trigger inflammatory responses and lead to cell death [39]. Huoxin Pill, a traditional Chinese medicine, can remarkably alleviate cardiac inflammation via repressing the TLR4/NF-κB signaling pathway [40]. Studies have also found that mediating TLR4/NF-κB signaling pathway can regulate antioxidant and anti-inflammatory activities to protect mice against AKI [41]. In addition, pyranochalcone-derived 5b has an obvious renoprotective effect on LPS-induced AKI, which is related to the inhibition of the TLR4/NF-κB signaling pathway [42]. In the study, mimics miR-22 could remarkably downregulate the LPS-induced increased TLR4, TLR2, MyD88, and p-NF-κB p65 protein expression, which indicated that miR-22 may alleviate sepsis-induced AKI by targeting HMGB1/TLR4/NF-κB signaling pathway.

The limitation of the study is that we did not use antagonist of the HMGB1/TLR4/NF-κB signaling pathway to further verify the role of miR-22 in AKI. In our future study, we will apply the antagonist of the HMGB1/TLR4/NF-κB signaling pathway to better verify the results of the study.

In conclusion, this study found that the expression of miR-22 was down-regulated while the expression of HMBG1 was up-regulated in the sepsis-related AKI model. Overexpression of miR-22 could target and suppress the expression of HMGB1, inhibit the release of inflammatory factors and the expression of HMGB1/TLR4/NF-κB signaling pathway-related proteins, to attenuate the sepsis-induced AKI. These data indicate that miR-22 may serve as a new treatment target in sepsis-induced AKI.

## Data Availability

All data are included in the manuscript.
